# Muscle oxidative stability, fatty acid and amino acid profiles, and carcass traits of broiler chickens in comparison to spent laying hens

**DOI:** 10.3389/fvets.2022.948357

**Published:** 2022-08-09

**Authors:** Mahmoud S. El-Tarabany, Omar A. Ahmed-Farid, Salah M. El-Bahy, Mohamed A. Nassan, Ayman S. Salah

**Affiliations:** ^1^Department of Animal Wealth Development, Faculty of Veterinary Medicine, Zagazig University, Zagazig, Egypt; ^2^Physiology Department, National Organization for Drug Control and Research (NODCAR), Cairo, Egypt; ^3^Department of Chemistry, Turabah University College, Taif University, Taif, Saudi Arabia; ^4^Department of Clinical Laboratory Sciences, Turabah University College, Taif University, Taif, Saudi Arabia; ^5^Department of Animal Nutrition and Clinical Nutrition, Faculty of Veterinary Medicine, New Valley University, El Kharga, Egypt

**Keywords:** spent laying hens, meat quality, carcass, nutritive value, oxidative stability, broilers

## Abstract

This research compared muscle oxidative stability, meat composition, and carcass traits in commercial broilers and spent laying hens. At week 65 of age, 40 ISA Brown laying hens were randomly selected to create 10 replicate cages (4 birds per cage). Also, 60 day-old Ross chicks were equally divided into six replicates (10 chicks each). Broiler chickens had a higher dressing percentage than spent hens (*P* = 0.027), but a lower abdominal fat percentage (*P* = 0.009). Spent hens had higher level of malondialdehyde (MDA) in the breast muscles (*P* = 0.001). Meanwhile, the MDA levels in thigh muscles did not differ in both groups (*P* = 0.328). Broiler chickens showed greater concentrations of saturated fatty acids (palmitic and stearic) in the breast (*P* = 0.012 and 0.006, respectively) and thigh (*P* = 0.033 and 0.038, respectively) muscles as compared to spent hens. Meanwhile, broiler chickens had lower concentrations of palmitoleic, oleic and eicosapentaenoic in the breast muscles (*P* = 0.002, 0.004 and 0.001, respectively). Also, spent hens had greater concentrations of linoleic in the breast and thigh muscles (*P* = 0.018 and 0.035, respectively). When compared to broiler chickens, spent hens had greater essential amino acids (isoleucine, methionine and tyrosine) concentrations in the breast muscles (*P* = 0.002, 0.001 and 0.036, respectively). Finally, while broiler chickens had superior carcass traits, spent hens showed better meat composition (higher polyunsaturated fatty acids and essential amino acids). Furthermore, the oxidative stability of the breast muscles of spent hens was lower than that of broilers. Spent hens can be used as an attractive source of chicken meat if certain precautions are adopted.

## Introduction

Chicken meat is in increased demand around the world due to its low fat and cholesterol content, as well as its affordable cost (1). Regular dietary intake of chicken meat has been recommended to prevent the prevalence of various diseases and have a good effect on human health due to its balanced nutritional components (2). According to Liu et al. (3), per capita consumption of chicken meat has grown five folds over the last four decades. Furthermore, there are no cultural or religious restrictions on the consumption of poultry products (4). Broilers, who are specifically developed for meat production, and layer hens, which lay eggs for human consumption, are the two main types of commercial chickens (5). With the rising demand for chicken meat, farm households are increasingly producing more broilers, especially on a large scale, because they can be shipped to market in 5 to 6 weeks, resulting in additional cost savings (6). Their meat has a great nutritional value, a soft texture and a pleasant flavor, and it is quite inexpensive (7).

Commercial laying hens worldwide outlast their productive lives between the ages of 60 and 72 weeks (8). Spent laying hens (SH) are female birds that have completed their egg-laying cycle, and their meat has a nutritional value comparable to that of commercial broilers, as well as being a rich source of protein (9). Furthermore, due to a decline in egg production rate, the number of SH increases with animal age. Meanwhile, the condemnation of spent laying hens (SH) could result in financial losses for farmers as well as major environmental issues (10). The myofibrillar protein and omega-3 fatty acid content of spent laying hens are high, but the edible meat quantity is low, and the sensory qualities are poor (11). Because the hens are slaughtered at an old age, their meat contains heat-stable cross-linked connective tissues that require a high level of heat to degrade (12). Two aspects, age and genetics, affect muscle composition of spent hen meat, which affects the meat's eating quality (2). Thus, despite their potential as protein sources, spent laying hens' low market value is due to their poor organoleptic qualities (13).

Meat quality, which is determined by a variety of characteristics such as genetics, age, body weight, and other environmental conditions, determines consumer acceptance (14). Consumer perception of meat and meat products has generally been found to be mainly influenced by nutrition value and physicochemical attributes. Although the physicochemical characteristics of meat are equally crucial for processing and customer acceptance, nutritional value is crucial. Both commercial layer stocks and broiler flocks are regularly farmed in Egypt. Commercial broilers have only been raised for about 40 days, yet they are now the primary variety of chicken sold in Egyptian markets. Another potential source of chicken meat is spent hens, which are aged chickens with meat that is extremely rough due to high heat-stable collagen levels (15). In previous investigations, genetics was identified as the most important aspect influencing chicken meat quality (16). Many researches on the meat quality of chicken breeds have focused on carcass traits and physicochemical characteristics (17). However, little information about the nutritional values (such as amino acid and fatty acid composition) of different varieties of chicken meat is published (18). Thus, the objective of this study was to compare muscle oxidative stability, meat composition and carcass traits in commercial broilers and spent laying hens.

## Materials and methods

### Chickens, management and experimental design

A commercial flock (ISA Brown laying hens) was closely observed at week 65, and 40 laying hens were randomly selected to create 10 replicate cages (four birds per cage). The laying hens were housed in conventional wire cages (50 cm x 46 cm x 42 cm, L x W x H), with 16 h as a daily light regimen. The temperature and relative humidity were both kept at recommended levels (24.0°C and 62%, respectively). The birds had free access to water and fed *ad libitum*. In addition, a standard corn-soybean layer diet was supplied to all laying hens (Table 1). The average hen-day egg production was 67 percent at the end of the trial period (week 72 of age).

**Table 1:** Ingredient composition and calculated chemical analysis of the basal diets for broiler and laying hens.

	**Starter broiler diet**	**Grower-Finisher broiler**	**Laying hens diet**
	**(1–21 d, g kg^−1^ DM basis)**	**diet (22–42d, g kg^−1^ DM basis)**	**(g kg^−1^ DM basis)**
**Ingredients**			
Yellow maize	605.0	650.0	602.0
Soybean meal (48%)	308.0	250.0	260.0
Corn gluten (60%)	40.0	35.0	-
Maize oil	-	18.0	25
Di- calcium phosphate	23.0	23.0	17
Limestone	14.0	14.0	87
DL- methionine	1.0	1.0	1.3
Lysine	1.0	1.0	-
**Vitamin and trace mineral mx**	3.5	3.5	3.0
Salt (NaCl)	3.5	3.5	2.4
**Sodium bicarbonate**	-	-	2.3
Coccidostate	1.0	1.0	-
**Calculated analysis**	
^a^ME (KJ / kg)	12,342	12,949	12,029
Crude protein	224.0	197.5	166.0
Calcium	10.5	10.5	37.7
Available phosphorus	4.5	4.5	4.5
Lysine	11.8	11.4	8.5
Methionine	4.8	4.5	3.9

a *ME, metabolizable energy*.

A local hatchery provided sixty day-old Ross chicks, which were equally divided into six replicates (10 chicks each). The initial stocking density was 15 chicks per square meter. Fresh wood shavings were supplied to the pens, and feed was *ad libitum* during the whole experimental period (42 days). The initial temperature was 34 °C, which progressively decreased to the comfort zone by day 21. Regular vaccinations for Newcastle disease (7^th^ and 18^th^ day of age) and Gumboro disease (D78, 14^th^ day of age) were administered as part of a routine program. To meet the standard requirements of broiler chickens, the birds were fed corn-soybean diets (Table 1).

### Body weight and carcass traits

Two spent laying hens were chosen from each replicate cage (*n* = 20) weighed, and slaughtered by the end of week 72. In addition, three female broiler chickens (*n* = 18) were picked from each pen (day 42 of age), weighed, and slaughtered. The spent laying hens were slaughtered using the HALAL method (19). After evisceration and removal of internal organs, carcasses were allowed to drain for 5 min before being cooled for 30 min at 2°C. The dressing percentage was estimated as the weight of the carcass in relation to the live BW. Following that, the abdominal fat pad (fat around the abdominal organs) and internal organs (heart, liver, and gizzard) were weighed and their percentages in relation to the live BW were calculated.

### Muscle fatty acid and amino acid profiles

The breast and thigh muscles were dissected from cooled carcasses (spent chickens and broilers) and connective and fatty tissues were carefully removed. The collected samples were used to conduct chemical analysis of muscle fatty acids (FA) and amino acids (AA) profiles. Lipids were extracted using a chloroform:methanol solution (2 mL:1 mL, vortexed for 2 min, and centrifuged at 1,792 g for 10 min). Following the esterification process, fatty acid methyl esters (FAME) were produced from the supernatant using methanol:sulphuric acid mixture (95 mL:5 mL) (20). Sigma-Aldrich provided the free FA (Cat. No. 24073, Sigma-Aldrich, St. Louis, MO, USA), and gas chromatography was used to analyze the samples (Agilent Technologies; 7890A, Santa Clara, CA, USA). The ideal flow rate was 1.0 mL/min for nitrogen as the carrier gas (21). The Supelco GC columns were used (SP2330 column; 30 mm × 0.32 mm × 0.2 μm film thickness; Supelco Analytical, Bellefonte, USA). With hydrogen as the carrier gas, the split model was adjusted at a gradient temperature protocol. Using an Agilent ChemStation system, the peaks of FAME were recognized by comparing them to the retention period of FA standards (Hewlett-Packard ChemStation, Wilmington, DE, USA). The concentrations of saturated fatty acids (SFA) and poly unsaturated fatty acids (PUFA) in breast and thigh muscles were expressed as g 100 g^−1^.

The modified procedure of Hughes et al. (22) was used to assess the contents of free AA in the breast and thigh muscles. Derivatization began with the generation of free AA from the intact muscles through the use of complete hydrolysis with HCl 6N for 2 h. Methanol was used as the derivatizing agent: TEA: deionized water: phenylisothiocyanate mixture (7 mL:1 mL:1 mL:1 mL). In order to separate and quantify the free AA by the Agilent HPLC (1200 series apparatus, Santa Clara, CA, USA), the samples and AA standards were loaded into the Nova-PakTM C18 column (4 μm, 3.9 x 4.6 mm). The flow rate was kept constant at 2 mL/min, the column temperature was set to 50°C, and the UV detector was set at a wavelength of 254 nm. The concentrations of essential amino acids (EAA) and non-essential amino acids (NEAA) in breast and thigh muscles were expressed as g 100 g^−1^.

### Muscle malondialdehydes

To assess malondialdehydes (MDA) concentrations, breast and thigh muscle samples were homogenized in a standard buffer solution, centrifuged at 700 × g, and the clear supernatant layer was collected (23). The samples were analyzed using the Agilent HPLC apparatus (Agilent HP 1100 series, USA). Supelcosil C18 was used as the analytical column (particle size of 5 m and a pore size of 8 nm). The detection wavelength was 250 nm and the flow rate was 1.5 ml/min.

### Statistical analysis

The IBM SPSS software package (Version 16.0; IBM Corp., NY, USA) was used to evaluate the provided data using the independent sample student (T) test. The standard error of the mean is shown for each of the results (SEM).

## Results

Carcass traits of broiler chickens and spent hens are illustrated in Table 2. Broiler chickens had greater dressing percentage than spent hens (P= 0.027). Meanwhile, broiler chickens had lower percentages of abdominal fat, liver and heart than spent hens (P=0.009, 0.013 and 0.001, respectively).

**Table 2:** Carcass traits of broiler chickens and spent laying hens.

**Parameter**	**Experimental groups**		
	**^a^BC**	**^b^SH**	**^c^SEM**	***P*-value**
Live weight (g)	1,942	1,867	22	0.105
Dressing (%)	72.1	69.3	0.74	0.027
Abdominal fat (%)	0.81	3.59	0.24	0.009
Liver (%)	2.23	2.45	0.06	0.013
Heart (%)	0.39	0.64	0.03	0.001

a *Broiler chickens*;

b *Spent laying hens*;

c *Standard error of mean*.

Fatty acids profile of breast and thigh muscles in broiler chickens and spent hens are described in Table 3, 4. Compared to broilers, spent hens showed greater concentrations of PUFA in the breast and thigh muscles (*P* = 0.001 and 0.037, respectively). Broiler chickens showed greater SFA (palmitic and stearic) concentrations in the breast and thigh (*P* = 0.033 and 0.038, respectively) muscles as compared to spent hens. Meanwhile, Broiler chickens had lower concentrations of palmitoleic, oleic and eicosapentaenoic in the breast muscles (*P* = 0.002, 0.004 and 0.001, respectively). Also, spent hens had greater concentrations of linoleic in the breast and thigh muscles (*P* = 0.018 and 0.035, respectively). The concentrations of α-Linolenic in the breast and thigh muscles did not differ in both groups (*P* = 0.350 and 0.523, respectively).

**Table 3:** Fatty acids profile (g 100 g^−1^) of breast muscle in broiler chickens and spent laying hens.

**Fatty acids**	**Experimental groups**		
	**^a^BC**	**^b^SH**	**^c^SEM**	***P*-value**
Myristic (C_14:0_)	0.868	0.872	0.03	0.685
Palmitic (C_16:0_)	33.7	27.7	1.85	0.012
Stearic (C_18:0_)	12.9	10.2	0.58	0.006
Myristoleic (C_14:1_)	1.02	0.79	0.04	0.022
Palmitoleic (C_16:1_)	1.07	1.35	0.06	0.002
Oleic (C_18:1_)	19.8	23.2	0.94	0.004
Linoleic (C_18:2n6_)	14.1	18.7	0.73	0.018
α-Linolenic (C_18:3n3_)	0.907	0.880	0.02	0.350
Eicosapentaenoic (C _20:5n3_)	0.625	0.735	0.03	0.001
Docosahexaenoic (C_22:6n3_)	0.527	0.507	0.02	0.508
^d^SFA	47.43	38.74	0.76	0.001
^e^PUFA	16.18	20.86	0.44	0.001

a *Broiler chickens*;

b *Spent laying hens*;

c *Standard error of mean*;

d *saturated fatty acids*;

e *polyunsaturated fatty acids*.

**Table 4:** Fatty acids profile (g 100 g^−1^) of thigh muscle in broiler chickens and spent laying hens.

**Fatty acids**	**Experimental groups**		
	**^a^BC**	**^b^SH**	**^c^SEM**	***P*-value**
Myristic (C_14:0_)	1.21	0.98	0.03	0.018
Palmitic (C_16:0_)	31.2	27.6	1.39	0.033
Stearic (C_18:0_)	11.6	10.2	0.59	0.038
Myristoleic (C_14:1_)	0.95	0.68	0.05	0.028
Palmitoleic (C_16:1_)	1.80	1.54	0.09	0.061
Oleic (C_18:1_)	25.2	23.8	1.63	0.399
Linoleic (C_18:2n6_)	17.2	20.3	1.52	0.035
α-Linolenic (C_18:3n3_)	0.618	0.597	0.02	0.523
Eicosapentaenoic (C _20:5n3_)	0.713	0.768	0.03	0.279
Docosahexaenoic (C_22:6n3_)	0.431	0.450	0.02	0.518
^d^SFA	44.01	38.65	0.94	0.011
^e^PUFA	18.83	21.98	0.62	0.037

a *Broiler chickens*;

b *Spent laying hens*;

c *Standard error of mean*;

d *saturated fatty acids*;

e *polyunsaturated fatty acids*.

Amino acids profile of breast and thigh muscles in broiler chickens and spent hens are described in Table 5, 6. When compared to broiler chickens, spent hens had greater EAA (isoleucine, methionine and tyrosine) concentrations in the breast muscles (*P* = 0.002, 0.001 and 0.036, respectively). Spent hens also had higher NEAA (serine, aspartic acid, glutamine, arginine, histidine and proline) concentrations in the breast muscles (*P * < 0.05). Meanwhile, with exception of threonine and alanine, the levels of amino acids in thigh muscles did not differ between the two groups (*P* > 0.05), with the exception of threonine and alanine.

**Table 5:** Amino acids profile (g 100 g^−1^) of breast muscle in broiler chickens and spent laying hens.

**Amino acids**	**Experimental groups**		
	**^a^BC**	**^b^SH**	**^c^SEM**	***P*-value**
^d^ **EAA**				
Lysine	6.70	6.95	0.21	0.472
Leucine	6.78	6.42	0.19	0.322
Isoleucine	2.91	3.39	0.09	0.002
Valine	3.59	3.79	0.12	0.243
Methionine	1.38	1.74	0.05	0.001
Threonine	3.17	3.13	0.08	0.774
Phenyl alanine	1.73	1.86	0.05	0.175
Tyrosine	2.06	2.32	0.06	0.036
^e^ **NEAA**				
Serine	2.38	2.87	0.08	0.001
Aspartic acid	7.63	8.82	0.32	0.004
Glutamine	10.34	12.64	0.42	0.009
Alanine	4.69	4.64	0.18	0.828
Arginine	4.42	5.03	0.20	0.020
Histidine	2.46	3.17	0.09	0.001
Glysein	4.39	4.71	0.13	0.128
Proline	1.30	1.62	0.05	0.016

a *Broiler chickens*;

b *Spent laying hens*;

c *Standard error of mean*;

d *Essential amino acids*;

e *Non-essential amino acids*.

**Table 6:** Amino acids profile (g 100 g^−1^) of thigh muscle in broiler chickens and spent laying hens.

**Amino acids**	**Experimental groups**		
	**^a^BC**	**^b^SH**	**^c^SEM**	***P*-value**
^d^ **EAA**				
Lysine	6.59	5.73	0.22	0.076
Leucine	6.28	5.21	0.24	0.062
Isoleucine	2.76	2.71	0.07	0.763
Valine	3.61	3.10	0.12	0.090
Methionine	1.39	1.46	0.05	0.480
Threonine	3.08	2.60	0.11	0.047
Phenyl alanine	1.67	1.55	0.06	0.341
Tyrosine	2.01	1.93	0.08	0.461
^e^ **NEAA**				
Serine	2.36	2.38	0.07	0.931
Aspartic acid	7.07	7.30	0.22	0.606
Glutamine	10.14	10.45	0.41	0.520
Alanine	4.70	3.80	0.15	0.046
Arginine	4.27	4.25	0.17	0.912
Histidine	2.43	2.64	0.11	0.317
Glysein	4.38	3.86	0.18	0.066
Proline	1.30	1.36	0.04	0.650

a *Broiler chickens*;

b *Spent laying hens*;

c *Standard error of mean*;

d *Essential amino acids*;

e *Non-essential amino acids*.

**Figure 1:**
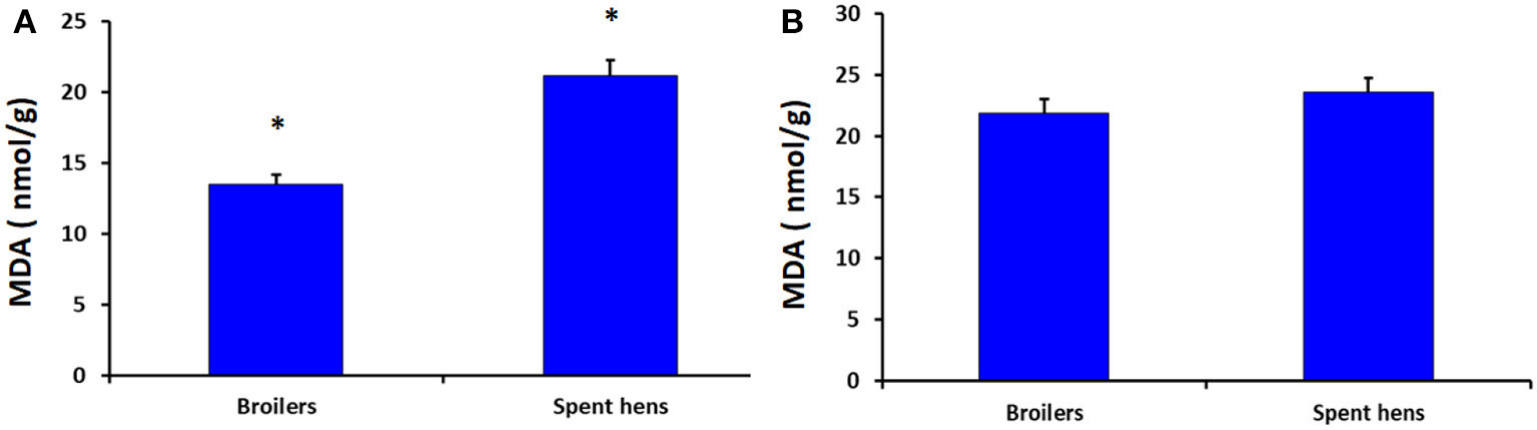
The concentrations of malondialdehyde (MDA) in the breast **(A)** and thigh **(B)** muscles (*P* = 0.001 and 0.328, respectively) of broiler chickens and spent laying hens. * Significant at level *P* < 0.05.

Figure 1 shows the levels of MDA in the breast and thigh muscles of broiler chickens and spent hens. Compared with broiler chickens, spent hens had higher level of MDA in the breast muscles (*P* = 0.001). Meanwhile, the MDA levels in thigh muscles did not differ in both groups (*P* = 0.328).

## Discussion

Food's functional role and characteristics have recently received increased attention, with the aim of improving consumer health and preventing diseases related to poor nutrition. The current study compared carcass characteristics, muscle oxidative stability, and meat composition in commercial broilers and spent ISA-Brown laying hens in order to see if spent laying hens could be used as a commercial source of poultry meat. In the current study, broiler chickens had greater dressing percentage, but lower abdominal fat percentage when compared to spent hens. The higher liver percentage in spent hens may re?ect excessive deposition of fat and elevated rate of lipogenesis. In accordance with these findings, Mueller et al. (24) found that Ross PM3 broiler chickens had higher dressing percentage, but lower abdominal fat percentage when compared with Lohman Dual chickens. Also, the dressing percentage in Ross 308 broilers was greater than that in Rhode Island Red (RIR) layers (25). They also demonstrated lower abdominal fat in Ross broilers as compared with RIR layers. In this study, the dressing percentage in broilers chickens (72.1%) was quite near to that reported in other studies (24, 25). At the same age, the dressing percentage in spent hens (69.3 %) was close to that reported in Mos spent hens (69.8 %), but higher than that of ISA Brown spent hens (63.0 %) (26). Genetic differences and feed regiment could be to responsible for this discrepancy. In this regard, despite the reduced dressing percentage expected in spent hens, ISA Brown spent hens achieved similar results to several dual-purpose breeds (27). On the contrary, no carcass yield differences were detected between commercial broiler breeders (week 60 of age) and Hubbard broiler chickens (28). Fat deposition was one significant difference. The fat content of the spent hens was definitely higher, as evidenced by the percentage of abdominal fat (almost four times). Similarly, Kaewkot et al. (25) found that RIR chickens had much (*P* < 0.05) abdominal fat than the Ross broiler chickens. In contrast, research by Tang et al. (17) and Mueller et al. (24) indicated that the proportion of abdominal fat in laying hens was much lower than that of indigenous chickens. It appears that genetic differences across chicken types, rather than specialization for laying or fattening, are the primary cause of variation in fat deposition.

The overall quality of meat is influenced by a number of factors. Differences in breeding purposes and animal age are among the primary players between broilers and spent hens. It's worth noting that a direct comparison of meat quality between broilers and spent hens is likely to be obscured by these, as well as other external factors like feeding regimen and rearing management. However, the broiler samples used in this study were originally intended to act as a baseline for determining the muscle composition of spent hens. Palmitic (C16:0), oleic (C18:1), and linoleic acids (C18:2:n-6) were the predominant fatty acids found in chicken meat from broilers and spent hens, as expected. They accounted for almost 75% of total fatty acids, which is consistent with earlier research findings (6, 18). Broiler chickens had greater concentrations of SFA in the breast and thigh muscles as compared to spent hens. In accordance with these results, Kaewkot et al. (25) discovered that Ross broilers had higher levels of SFA in the breast and thigh muscles when compared to the Pradu Hang Dam and RIR chickens. Chen et al. (2) also demonstrated that SFA levels in Arbor Acres broilers breast and thigh muscles were comparable to those found in Hyline brown laying hens. According to Jayasena et al. (6), total SFA contents of breast muscles were not significantly different between broilers and spent hens. In the meantime, they observed that the myristic acid level of breast meat in spent hens was significantly higher than in commercial broilers, contradicting the findings of this study.

In the current study, the breast meat lipids of spent hens were much richer in PUFA (linoleic and eicosapentaenoic) than those of the broiler chickens; at the expense of SFA. These changes in the fatty acid composition may influence the sensory quality of the meat, in addition to being important for human health (29), with the n-3 PUFA being favored over other fatty acids. This may be a reflection of the physiological immaturity of broilers when they are slaughtered, and it is possible that the adult birds (spent hens) might show a difference in their response to dietary fatty acids (30). Furthermore, the levels of dietary fatty acids of standard broiler diets are greatly differed than commercial laying hen diets. Indeed, the vast majority of table chickens are slaughtered before they reach puberty, while spent hens are full mature, and thus such differences are likely to be of practical importance. Consistent with these findings, Chen et al. (2) reported a tendency of linoleic acid to be significantly higher in both breast and thigh muscles of Hyline laying hens when compared to Arbor Acres broilers. Choo et al. (31) also noticed that the PUFA content of breast meat in egg-type chickens was higher (*p* < 0.05) than in Ross broilers., including essential fatty acids such as linoleic acid (C18:2 ω6). Similar results were also reported in spent (18-month-old) White Leghorn laying hens (32). Indeed, the PUFA contents of spent hens' breast and thigh muscles were much lower than that of aged broiler chickens (33) or dual-purpose birds (34). Eicosapentaenoic acid, as a typical n-3 FA in marine fish (35), was much higher in the breast muscles of spent hens than in broiler chickens despite the minor amount. In contrast to the current findings, Tangnet al. (17) found that the indigenous chicken types had equal or even lower PUFA contents in the intramuscular lipids. In a more recent research, Bongiorno et al. (36) also revealed similar PUFA contents in the breast and thigh muscles of indigenous Italian chicken breeds. Given that poultry meat is one of the main sources of PUFA in developed economies (30), spent laying hens might be used as a low-cost, moderate-quality meat source.

Lipid peroxidation degrades meat quality and frequently dictates the shelf life of chicken meat (37). In this context, high PUFA fraction of the lipids in meat greatly increases the potential of fatty acid oxidation, reducing the meat's shelf life and increasing the risk of chemicals harmful to human health (38). Indeed, the oxidative stability of unsaturated lipids decreases as their degree of unsaturation increases, and so poultry meat with an enhanced PUFA contents will be more susceptible to oxidation. Lipid peroxidation also results in off-flavor, off-odor, discoloration, texture changes and a relative loss of nutritional value (39). Apart from the lipids themselves, there may be genetic differences in meat components that affect lipid oxidation. Endogenous catalysts such as myoglobin, free ionic iron, reducing chemicals and catalase are among them (40). In comparison to Ross broilers, spent hens had much higher levels of PUFA and MDA in the breast muscles, suggesting that the oxidative shelf life of the spent hens' breast muscles may be shorter than that of the broiler meat. In this context, the exact reasons for the variation in lipid oxidation identified in this study between spent hens and broilers chicken remain unknown. Consistent with these findings, Dalle Zotte et al. (41) found that meat from indigenous Italian chickens exhibited greater levels of oxidation than that in commercial broiler chickens. On the contrary, Castellini et al. (42) found lower extent of lipid oxidation in indigenous chickens compared with Ross chickens. Meanwhile, others did not report significant differences in muscles MDA levels betwwn RIR chickens and Ross broilers (25). The discrepancy in muscle MDA levels between studies could be due to various factors, including the nutritional regimen and the analytical method (43).

The composition and quantity of amino acid content in chicken meat is another factor to consider. However, it is necessary to determine which amino acid is the most relevant (44). In the present study, spent hens had greater concentrations of EAA (isoleucine, methionine and tyrosine) in the breast muscles as compared to broiler chickens. Similarly, indigenous Italian chickens meat exhibited greater contents of essential (isoleucine, leucine and threonine) and non essential (glycine and proline) amino acids than commercial broiler chickens (41). Dalle Zotte et al. (45) further confirmed that the breast meat of Polverara chickens had a higher content of all amino acids than hybrid chicken meat. Protein digestibility and deposition are affected by factors such as age, and the amino acid composition of meat is known to be affected by diet (46). Some studies found no differences in amino acid profiles when comparing indigenous chicken breeds to commercial broilers (47).

## Conclusion

The findings of the current study confirm the difference in carcass traits and meat composition between spent hens and broiler chickens. Broiler chickens had a higher dressing percentage than spent hens, but a lower abdominal fat percentage. Spent hens showed lower SFA contents in their breast muscles, but higher PUFA and EAA contents than broiler chickens. Meanwhile, the breast muscles of spent hens had lower oxidative stability than broilers. if certain precautions are adopted, spent hens can be used as an attractive source of chicken meat. In view that the present research is a preliminary trial, the comparative studies should be expanded to include different chicken breeds and larger commercial flocks, as well as the relationship between muscle amino acids and physicochemical properties in aged chickens.

## Data availability statement

The raw data supporting the conclusions of this article will be made available by the authors, without undue reservation.

## Ethics statement

The animal study was reviewed and approved by the Animal Ethics Committee of Zagazig University, Egypt (Project Approval No. ZU-IACUC/2/F/94/2021).

## Author contributions

Conceptualization, methodology and data curation: OA-F, AS, and ME-T. Formal analysis, investigation, and supervision: OA-F, AS, SE-B, and ME-T. Resources, writing original draft preparation, and visualization: OA-F, AS, ME-T, and MN. Writing-review and editing: AS, SE-B, and ME-T. All authors contributed to the article and approved the submitted version.

## Conflict of interest

The authors declare that the research was conducted in the absence of any commercial or financial relationships that could be construed as a potential conflict of interest.

## Publisher's note

All claims expressed in this article are solely those of the authors and do not necessarily represent those of their affiliated organizations, or those of the publisher, the editors and the reviewers. Any product that may be evaluated in this article, or claim that may be made by its manufacturer, is not guaranteed or endorsed by the publisher.
